# EGFR kinase inhibitors and gastric acid suppressants in EGFR-mutant NSCLC: a retrospective database analysis of potential drug interaction

**DOI:** 10.18632/oncotarget.13458

**Published:** 2016-11-19

**Authors:** Nesaretnam Barr Kumarakulasinghe, Nicholas Syn, Yu Yang Soon, Atasha Asmat, Huili Zheng, En Yun Loy, Brendan Pang, Ross Andrew Soo

**Affiliations:** ^1^ Department of Haematology-Oncology, National University Cancer Institute, Singapore; ^2^ Cancer Science Institute of Singapore, National University of Singapore, Singapore; ^3^ Yong Loo Lin School of Medicine, National University of Singapore, Singapore; ^4^ Department of Radiation Oncology, National University Cancer Institute, Singapore; ^5^ Department of General Surgery, Tan Tock Seng Hospital, Singapore; ^6^ National Registry of Diseases Office, Health Promotion Board, Singapore; ^7^ Department of Pathology, National University Health System, Singapore

**Keywords:** gefitinib, erlotinib, drug-drug interactions, NSCLC, gastric acid suppression

## Abstract

**Background:**

Erlotinib and gefitinib are weak base drugs whose absorption and clinical efficacy may be impaired by concomitant gastric acid suppressive (AS) therapy, yet proton pump inhibitors (PPIs) and histamine-2 receptor antagonists (H2As) are widely indicated in non-small cell lung cancer (NSCLC) patients for the prevention and treatment of erlotinib-induced gastrointestinal injury and corticosteroid-associated gastric irritation. We assessed the clinical relevance of this potential drug-drug interaction (DDI) in a retrospective cohort of *EGFR*-mutant NSCLC patients.

**Results:**

The AS usage rate was 35%. In the overall cohort, AS users did not experience poorer OS (HR: 1.47, 95% CI: 0.92 – 2.35, *P* = 0.10; median, 11.4 versus 17.5 months) or PFS (HR = 1.37, 95% CI: 0.89 – 2.12, *P* = 0.16; median, 7.6 versus 8.7 months) compared with non-users in multivariate Cox regression analysis. However, subgroup analyses indicated that AS usage was associated with significantly poorer OS and PFS in patients who had fewer or milder comorbidities (Charlson comorbidity index ≤ 2), those with Karnofsky performance status < 90, and never-smokers.

**Materials and Methods:**

A retrospective database analysis of 157 patients given erlotinib or gefitinib for *EGFR*-mutant advanced NSCLC from two institutions was conducted. Patients were classified as AS-users if the periods of AS and anti-EGFR therapy overlapped by ≥ 30%. Overall survival (OS) and progression-free survival (PFS) were assessed according to AS usage.

**Conclusions:**

Concomitant AS therapy did not have an adverse impact on OS and/or PFS in the overall cohort. Our subgroup findings should be regarded exploratory and require replication in a large prospective cohort.

## INTRODUCTION

The epidermal growth factor receptor (EGFR) tyrosine kinase inhibitors (TKIs), gefitinib and erlotinib, are orally administered small-molecule targeted therapeutics recommended as front-line therapy for a subset of advanced non-small cell lung cancer (NSCLC) patients who harbour *EGFR*-sensitizing somatic mutations [1]. In selected patients, single-agent gefitinib or erlotinib in the first-line setting has demonstrated superiority over standard platinum-based doublet chemotherapy in large phase III randomized trials, yielding response rates of 62% – 83% and median progression-free survival duration of 9.2 – 13.1 months [2-6]. The main side-effects include rash, diarrhoea, anorexia and fatigue [7], which are generally well-tolerated and associated with lower rates of treatment-related drug discontinuation or dose modifications compared to platinum-containing regimens [4].

However, a more severe complication is the risk of gastrointestinal (GI) perforation among erlotinib-treated patients, which has led to fatalities [8] and the US Food and Drug Administration (FDA) revising the prescribing information in 2010 [9]. The pathophysiology of acute GI events may be related to higher levels of EGFR expression in the epithelium of the GI tract [10]. In a small post-marketing study of 103 erlotinib-treated NSCLC patients, the incidence of GI injury was 4.9% [11]. Permanent drug discontinuation is advised if it occurs, and the risk is higher in patients receiving concomitant non-steroidal anti-inflammatory drugs, corticosteroids, anti-angiogenic agents, taxane-based chemotherapy, and who have prior history of peptic ulceration or diverticular disease [9, 11]. Prophylactic acid suppression (AS) therapy, such as histamine-2 receptor antagonists (H2A) and proton pump inhibitors (PPI), may be given to patients receiving erlotinib [12]. AS are also widely used among the general population as over-the-counter medications to counter symptoms of gastroesophageal reflux disease, and market research also indicates that the prevalence of concurrent AS prescription is 33.2% – 46.3% among lung cancer patients in the United States [13].

AS therapy suppresses acid secretion by parietal cells and cause the intragastric pH to be raised from ~1.2 to ~4. PPIs have a longer lasting effect (~24 h) compared to H2As (~12 h) [14]. Over half of recently approved molecular targeted tyrosine kinase inhibitors, including erlotinib and gefitinib, exhibit weakly basic chemical properties and pH-dependent solubility [13, 15, 16]. The acid dissociation constants (pKa_1_) of erlotinib and gefitinib are both ~5.4 [13]. Thus, under hypochlorhydic conditions induced by AS, the equilibrium shifts from the ionized to the non-ionized form which is less readily absorbed. This pH-dependent drug-drug interaction (DDI) may diminish drug exposure and consequently, the clinical efficacy of erlotinib and gefitinib. Due to the extended period of gastric acid suppression, staggered dosing is unlikely to eliminate potential drug interactions, although it may be theoretically possible to minimise the interaction by prescribing H2As at night instead of the twice-daily regimens approximately 12 hours apart from the TKI.

The potential for an antagonistic drug interaction is reflected in the FDA boxed warnings to prescribing information for erlotinib and gefitinib [9, 17]. According to the package inserts, concomitant omeprazole 40 mg daily decreased erlotinib area under the concentration-time curve (AUC) and maximum plasma concentration (C_max_) by 46% and 61% respectively in healthy volunteers, while ranitidine 300 mg daily reduced erlotinib AUC and C_max_ by 33% and 54% [9]. High-dose ranitidine to maintain intragastric pH > 5.0 resulted in 44% and 70% reduction of gefitinib AUC and C_max_ respectively [17]. However, other studies demonstrate that concomitant use of AS do not appear to have an antagonistic interaction with TKI pharmacokinetics [18-21]. Of higher evidential value are a retrospective analysis of the BR.21 trial database and a population pharmacokinetic-pharmacodynamic (PKPD) study in Japanese NSCLC patients which did not find significant differences in erlotinib plasma concentrations and exposure respectively between AS users and non-users [19, 20].

Two retrospective analyses, including the BR.21 database review, have attempted to address whether AS therapy may compromise the clinical efficacy of erlotinib and gefitinib in advanced NSCLC patients [20, 22]. Of note, in both these studies patients were unselected for activating *EGFR* mutations. Hilton et al. reported a lack of significant differences in progression free survival (PFS) and overall survival (OS) in AS users and non-users [20], whereas Chu et al. reported poorer median PFS (1.4 vs 2.3 months, *P* < 0.001) and OS (12.9 vs 16.8 months, *P* = 0.003) in AS users vs non-users [22]. A potential source of study heterogeneity is the underlying difference in proportions of *EGFR* wild-type and mutant patients in each cohort, whereby the number of *EGFR*-mutant patients was unknown in the former study while only 4/124 patients in the latter study harboured *EGFR* mutations.

Since the *EGFR* mutational status may confound attempts to address whether AS therapy adversely impacts PFS and OS in erlotinib or gefitinib-treated NSCLC patients, we performed a retrospective study examining a consecutive series of patients who tested positive for known activating *EGFR* mutations, who received EGFR TKIs with or without concomitant AS therapy.

## RESULTS

One hundred and ninety-one patients given erlotinib or gefitinib for *EGFR*-mutant non-small cell lung cancer were identified. Thirty-four patients were excluded from analyses as we were unable to determine the prescription dates when they initiated treatment with gefitinib or erlotinib. Hence, 157 patients were evaluable for overall and progression-free survival, of which, 55 patients had clinically significant concomitant PPI or H2A prescriptions (AS users; ≥ 30% overlap with the duration of anti-EGFR therapy). Baseline clinical characteristics and demographics were generally well-balanced with the exception of the presence of brain metastases and Karnofsky performance status (Table 1).

**Table 1:** Baseline clinical characteristics and demographics

	AS users (N = 55)	AS non-users (N = 102)	*P* value
**Age – yr**			0.85
Mean	61.7 ± 9.8	62.0 ± 10.8	
Range	39 – 83	30 – 86	
**Sex – no./total no. (%)†**			0.60
Male	27/48 (56.3)	48/93 (51.6)	
**Race – no. (%)**			0.62
Chinese	48 (87.3)	86 (84.3)	
Malay, Indian and Others	7 (12.7)	16 (15.7)	
**Karnofsky Performance Status – no. (%)**			<0.01
<90	28 (50.9)	27 (26.5)	
90 – 100	27 (49.1)	75 (73.5)	
**Charlson Comorbidity Index – no./total no. (%)†**			0.73
≤2	48/55 (87.3)	90/101 (89.1)	
3	7/55 (12.7)	11/101 (10.9)	
**Smoking history – no./total no. (%)**			0.16
Smoker or former smoker	6/43 (14.0)	22/89 (24.7)	
Never-smoker	37/43 (86.0)	67/89 (75.3)	
**Brain metastases – no. (%)**			<0.01
Present	34 (61.8)	28 (27.5)	
**Liver metastases – no. (%)**			0.84
Present	11 (20.0)	19 (18.6)	

† Data could not be obtained for some patients, hence the proportion is calculated using the number of evaluable patients as the denominator.

**Figure 1:**
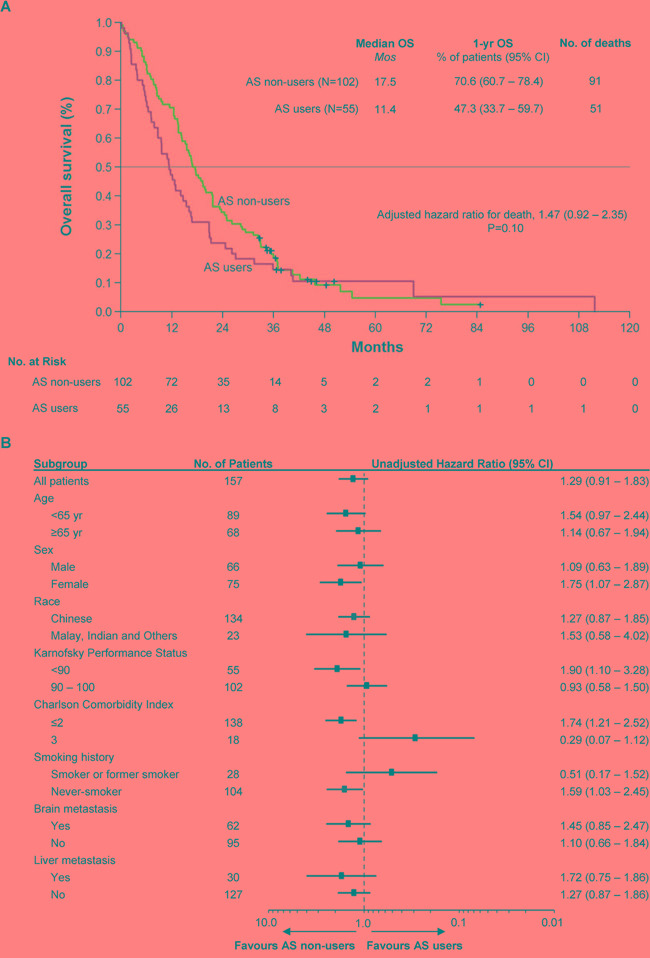
Kaplan-Meier Curve of Overall Survival in the Study Population and Forest Plot of Subgroup Analysis. **Panel A.** shows the Kaplan-Meier survival curves for AS users and non-users. The median OS was 11.4 months among AS users compared to 17.5 months among non-users (HR = 1.47, 95% CI: 0.92 – 2.35, *P* = 0.10). Overall survival was adjusted for baseline imbalances and all potentially prognostic clinical characteristics (including patient age, presence of brain metastases, presence of liver metastases, smoking history, race, sex, Karnofsky performance status and Charlson comorbidity index). **Panel B.** shows the heterogeneity of the treatment effect across clinical and demographic subgroups. In most cases, HRs were consistent with that of the overall cohort; however, the HR for death was increased in females, symptomatic patients (KPS < 90), those with milder or fewer co-morbidities (CCI ≤ 2), and never-smokers who received AS therapy compared to those who did not.

At the cutoff date for overall survival (OS) of June 15, 2016, which entails a median follow-up of 50.0 months and 48.2 months among AS users and non-users, 51 and 91 deaths respectively had occurred. Figure 1A shows the Kaplan-Meier survival curves for AS users and non-users. The median OS was 11.4 months among AS users compared to 17.5 months among non-users (unadjusted univariate HR = 1.29, 95% CI: 0.91 – 1.83, *P* = 0.15). Adjustment for baseline imbalances and all potentially prognostic clinical characteristics (which included patient age, presence of brain metastases, presence of liver metastases, smoking history, race, sex, Karnofsky performance status and Charlson comorbidity index) resulted in a more pronounced impact of AS therapy, with a HR of 1.47 (95% CI: 0.92 – 2.35), but without reaching statistical significance (*P* = 0.10; Table 2, multiple Cox regression model). The heterogeneity of the treatment effect was explored across patient subgroups based on baseline disease characteristics (Figure 1B). In most subgroups, HRs were consistent with that of the overall cohort; however, the hazard ratio for death was increased in females, symptomatic patients (KPS < 90), those with milder or fewer co-morbidities (CCI ≤ 2), and never-smokers who received AS therapy compared to those who did not.

**Table 2:** Multivariate Cox Regression Analysis for Overall Survival and Progression-Free Survival

Variable	HR	OS	*P*	HR	PFS	*P*
		**95% CI**			**95% CI**	
AS medication (yes *v* no)	1.47	0.92 – 2.35	0.103	1.37	0.89 – 2.12	0.155
Age (≥ *v* < 65 yr)	1.33	0.86 – 2.08	0.202	1.11	0.72 – 1.71	0.633
Sex (male *v* female)	1.06	0.66 – 1.72	0.796	1.03	0.65 – 1.62	0.914
Race (Malays, Indians and Others *v* Chinese)	1.22	0.68 – 2.17	0.508	0.79	0.44 – 1.39	0.410
Karnofsky Performance Status (90–100 *v* <90)	0.56	0.36 – 0.86	0.009	0.81	0.54 – 1.22	0.312
Charlson Comorbidity Index (3 *v* ≤2)	0.49	0.20 – 1.21	0.121	0.57	0.25 – 1.30	0.183
Smoking history (smoker or former smoker *v* never-smoker)	1.66	0.98 – 2.81	0.061	1.66	1.01 – 2.75	0.046
Brain metastasis (yes *v* no)	1.06	0.68 – 1.66	0.800	1.21	0.80 – 1.83	0.368
Liver metastasis (yes *v* no)	1.07	0.63 – 1.82	0.794	1.44	0.86 – 2.37	0.154

**Figure 2:**
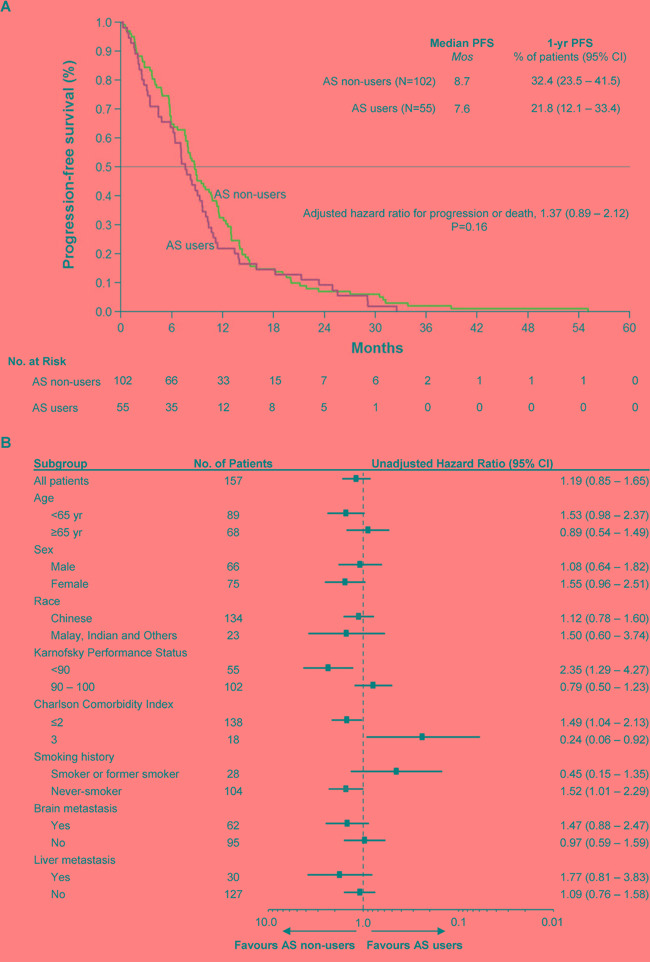
Kaplan-Meier Curve of Progression-Free Survival in the Study Population and Forest Plot of Subgroup Analysis. **Panel A.** shows the Kaplan-Meier survival curves for AS users and non-users. The median PFS was among 7.6 months among AS users compared to 8.7 months among non-users (HR = 1.37, 95% CI: 0.89 – 2.12, *P* = 0.16). There were no censored observations. Multivariate Cox regression was performed to adjust for baseline imbalances and potential confounding clinical variables, as in Figure 1. **Panel B.** shows the forest plot of the treatment effect in various patient subgroups. As in the case with overall survival, the hazard ratio for progression or death was increased among never-smokers, symptomatic from cancer (KPS < 90) or had fewer or milder co-morbidities (CCI ≤ 2).

In this cohort, the median progression-free survival (PFS) among AS users and non-users are 7.6 months and 8.7 months (Figure 2A; unadjusted univariate HR = 1.19, 95% CI: 0.85 – 1.65, *P* = 0.16). No observations were censored as all patients experienced either disease progression or death. Multivariate Cox regression accounting for baseline differences and prognostic factors yielded a modest increase in the impact of AS therapy (HR = 1.37, 95% CI: 0.89 – 2.12, *P* = 0.16). In subgroup analysis (Figure 2B), the effect of AS therapy on disease control varied depending on the presence of comorbidities, with patients having mild to moderate comorbidities (CCI ≤ 2) more likely to benefit from avoiding AS therapy, and patients at higher risk of mortality from other diseases (CCI = 3) less likely to be adversely affected by AS therapy. As in the case with overall survival, the hazard ratio for progression or death was increased among never-smokers, symptomatic from cancer (KPS < 90) or had fewer or milder co-morbidities (CCI ≤ 2) who received AS therapy compared to those who did not.

## DISCUSSION

The results of this study demonstrate that AS therapy, on whole, does not compromise the clinical efficacy of erlotinib and gefitinib: no appreciable differences in overall (HR of 1.47, 95% CI: 0.92 – 2.35, P = 0.10; Figure 1A, Table 2) and progression-free survival (HR = 1.37, 95% CI: 0.89 – 2.12, P = 0.16; Figure 2A, Table 2) were detected between AS users and non-AS users, corroborating the findings of Zenke and colleagues, who have conducted the only other retrospective study in a molecularly-selected, EGFRi-sensitive cohort [23]. However, our retrospective data further identifies patient subgroups defined by smoking history, performance status, sex and presence of comorbidities who may be more susceptible to clinically relevant antagonistic drug interactions (Figures 1B, 2B).

H2A and PPI medications are widely consumed by the general population and cancer patients for symptomatic relieve of gastroesophageal reflux disease, and are further commonly indicated during the course of erlotinib treatment to prevent GI injury [12, 13]. To our knowledge, this is the largest study to examine the potential pharmacological interaction between concomitant AS therapy and erlotinib or gefitinib specifically in *EGFR*-mutant non-small cell lung cancer patients.

Several reasons may account for the lack of antagonistic drug interaction in the overall cohort. Firstly, activating mutations in *EGFR* confer exquisite sensitivity to EGFR TKIs in non-small cell lung cancer, with cell lines harbouring these mutations being 10 to 50-fold more sensitive to gefitinib [24, 25]. Hence, despite reduced bioavailability, plasma levels of erlotinib and gefitinib may remain sufficient to achieve effective EGFR inhibition. Furthermore, there is no evidence that the steady state trough concentrations of erlotinib or gefitinib are reduced below the target concentration by AS use [20]. Secondly, the approved dosages of erlotinib and gefitinib are slightly above the biologically optimal dosage. For instance, although the approved dosage for gefitinib is 250 mg/day, early phase I and clinical pharmacodynamic studies actually indicated that the clinical and biological activity are only dose-dependent up to 150 mg/day, beyond which there are limited additional clinical benefits to reap [26, 27]. Similarly, a small retrospective study of 7 *EGFR*-mutant patients who were treated with erlotinib at the lowest available tablet size of 25 mg daily demonstrated a response rate of 71.5% and median PFS of 17 months (95% CI: 6-35 months), which is comparable with the approved dosage of 150 mg/day [28, 29].

Overall survival may be affected by many important parameters. In this study, significantly more AS users had brain metastases at baseline compared to non-users (Table 1). Corticosteroids such as dexamethasone are frequently given to control cerebral vasogenic edema and reduce neurologic symptoms secondary to brain metastases, which are common in advanced NSCLC, but in turn may necessitate AS therapy to reduce gastric irritation [30]. This underscores another factor accounting for the high prevalence of AS usage among cancer patients. However, in multivariate Cox analyses, the presence of brain metastasis was not found to independently impact overall and progression-free survival.

As with retrospective reviews, there are several limitations to consider. First, there were several clinical variables that we could not measure. We were unable to ascertain information on the frequency and severity of adverse events, which were not consistently recorded. Secondly, we were also reluctant to report response rates as long intervals between radiographic assessments hampered accurate assessment of the treatment responses. This recapitulates a common limitation of many retrospective analyses, whereby imaging assessments are less frequent and regular, and may not be standardised in terms of frequency, interval and follow-up protocol compared to prospective studies. Finally, we also acknowledge confounders such as patient compliance, dietary factors and over-the-counter usage of gastric acid suppressants, which could otherwise be controlled in a prospective trial. As such, our results should be regarded as exploratory and require replication in a large prospective cohort.

In summary, we have performed a comprehensive literature survey and retrospective evaluation of the DDI between erlotinib/gefitinib and H2A/PPI therapies. Based on the published literature as well as our own data, we conclude that whilst AS therapy is likely to modify the bioavailability of EGFR TKIs, there is limited evidence that the pharmacokinetic interaction adversely impacts the PFS and OS outcomes in *EGFR*-mutant NSCLC patients. Large prospective studies are warranted to confirm our findings, and meta-analyses should be performed when additional retrospective data become available. It is just as important to seek out potential pharmacological strategies to eliminate this pharmacokinetic interaction. For instance, van Leeuwen and colleagues recently reported that simply ingesting the acidic beverage Cola with erlotinib improves its bioavailability in patients who are also receiving esomeprazole [31]. Moreover, this study highlights the importance of dietary factors – which are hard to control – in influencing the absorption of oncological TKIs. We further caution that our results should not be extrapolated to other small-molecule oncological therapeutics such as crizotinib and sorafenib, many of which have weakly basic properties [13].

## MATERIALS AND METHODS

We identified patients who tested positive for known sensitizing *EGFR* mutations (EGFRmut^+^) between January 2008 and December 2013 from the Department of Molecular Testing, National University Health System's database. Included patients had been prescribed gefitinib or erlotinib for pathologically-confirmed advanced non-small cell lung cancer, and had detailed follow-up data available. Data was abstracted for the following clinical variables: treatment prescription dates, age, gender, race, smoking status, type of *EGFR* mutation, Charlson co-morbidity, Karnorfsky performance status, metastatic sites, date of diagnosis, last follow-up and, if deceased, cause of death. Clinically significant concomitant prescription of PPI or H2A (all generations) was defined as ≥ 30% overlap with the duration of anti-EGFR therapy. Approval was obtained from the local institutional review boards to perform this retrospective medical record review.

### Statistical analyses

Baseline differences in clinical characteristics and demographics were evaluated using Fisher's exact test or *χ* ^2^ test for proportions, and unpaired t-tests for continuous variables. Overall survival (OS) was calculated from the date of initial treatment with gefitinib or erlotinib until death. Progression-free survival (PFS) was defined as the time from treatment initiation until disease progression, clinical deterioration, or death. Patients without these events were censored on their last follow-up visit. PFS and OS were analysed by the Kaplan-Meier method, and treatment comparisons were by the log-rank test. The median follow-up duration of overall survival was estimated using the reverse Kaplan Meier [32]. Cox proportional hazards regression was used to adjust the treatment effect of AS therapy for other clinical variables, which included patient age, presence of brain metastases, presence of liver metastases, smoking history, race, sex, Karnofsky performance status and Charlson comorbidity index. The proportional hazards assumption was tested and confirmed for each of these variables using the method of Grambsch and Therneau [33]. *P* values of less than 0.05 (two-sided) were considered to indicate nominal statistical significance. STATA (version 13, StataCorp) was used for analyses.
